# Zygomatic Implants in a Patient With History of Mucormycosis: A Case Report

**DOI:** 10.7759/cureus.65512

**Published:** 2024-07-27

**Authors:** Dhanashree A Minase, Seema Sathe, Anjali Borle, Bhushan P Mundada, Khushbu Doshi

**Affiliations:** 1 Prosthodontics, Sharad Pawar Dental College, Datta Meghe Institute of Higher Education and Research, Wardha, IND; 2 Oral and Maxillofacial Surgery, Sharad Pawar Dental College, Datta Meghe Institute of Higher Education and Research, Wardha, IND

**Keywords:** zygoma, implants, mucormycosis, covid, post covid mucormycosis

## Abstract

Exposure to mucor mold can lead to mucormycosis, a rare fungal infection with severe consequences, especially in immunocompromised individuals. The year 2020 highlighted the need for orofacial rehabilitation, presenting challenges in cases of maxillary absence. This case report details zygomatic implants, which offer a promising solution by providing stable support even in the absence of significant bone mass due to surgical excision post-COVID-19 mucormycosis. Zygomatic implants outperform traditional grafting techniques. The atrophic or resected jaws can be effectively restored with a single-piece zygomatic implant, which has the lowest rate of postoperative problems, including bone loss, mucositis, peri-implantitis, and screw loosening or fractures. In this case report, four zygomatic implants were placed in the zygomatic bone, and immediate loading was performed. Restoration of patient aesthetics, phonetics, and functional masticatory efficiency were restored.

## Introduction

Exposure to mucor mold, which is frequently found in plants, soil, decomposing fruits, manure, and vegetables, the air, and even in the mucous of healthy individuals, can result in a rare fungal infection known as mucormycosis. It affects the sinuses, brain, and lungs, and in those with diabetes or serious immunocompromised people, it can be fatal. The year 2020 was a disastrous year for world health because of a rare virus that spread quickly around the globe and quickly became one of the leading causes of death, exposing the shortcomings of the healthcare systems [1,2]. As a result, a large number of people in various age groups needed orofacial rehabilitation [3]. Due to the absence of the maxilla, which limits the alternatives available for rehabilitation, this might be a difficult undertaking. Dentition is being restored in these individuals using a variety of techniques, including zygomatic implants, basal implants, and patient-specific implants [4], which reduce the requirement for grafting surgeries in the edentulous maxilla [5-7]. However, the relevance of these structures' support during the zygomatic implant placement process for rehabilitation was questioned in these individuals due to the whole or partial loss of maxilla and/or palatal bone. For edentulous patients lacking sufficient bone mass for dental implants, the zygoma implant was created [8]. This case report shows four zygomatic implants that were placed in the zygomatic bone as the bone quality was not sufficient in the maxillary arch for endosteal implants followed by the prosthesis insertion.

## Case presentation

A 49-year-old female patient was referred to the prosthodontic department. For the past two to three weeks, the patient reported experiencing discomfort, a persistent headache, and foul-smelling nasal discharge. The patient's previous history of COVID-19 infection was verified by a biopsy, which supported the mucormycosis diagnosis. 

Under general anesthesia, the patient underwent a maxillectomy, which compromised both function and appearance by removing the maxillary dentition. Following this treatment, the defect was addressed, and the restoration of function and aesthetics was facilitated by the strategically placed quad zygomatic implants.

**Figure 1:**
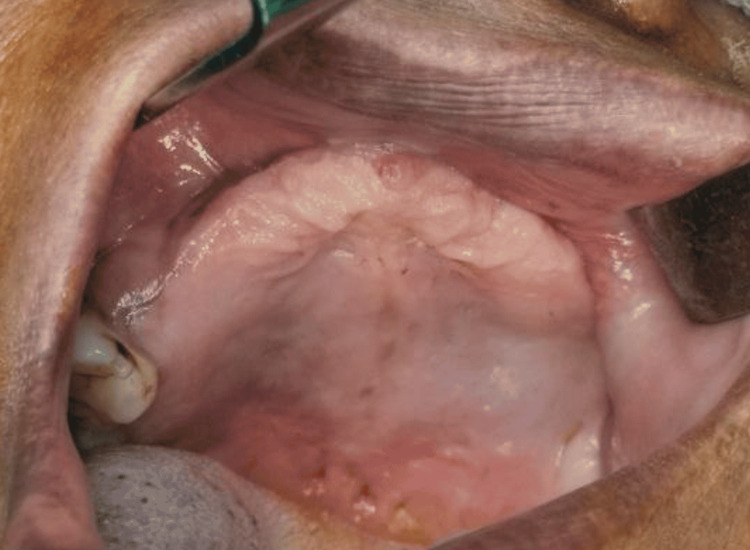
Edentulous maxillary arch with only 16, 17 teeth present

**Figure 2:**
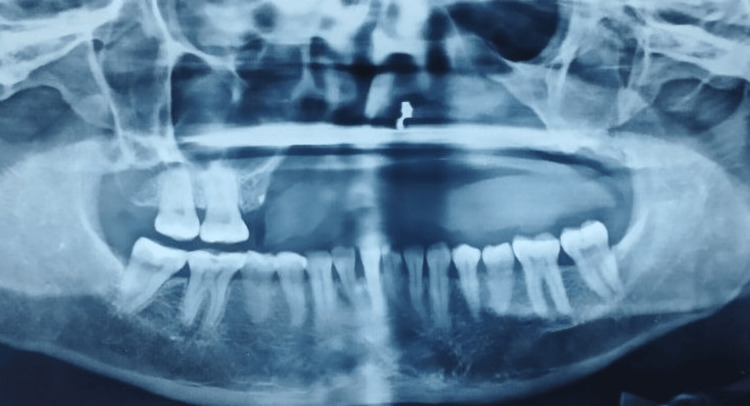
Radiograph showing less amount of bone in the maxilla

Upon extraoral inspection, there was a significant loss of support for the upper lip, poor facial aesthetics, and difficulty speaking. The temporomandibular joint was bilaterally synchronous with no crepitus, or clicking sound. The mandibular occlusal plane and the dentition were not deranged because it was a case of immediate restoration following maxillectomy. The nature of the defect, the patient's financial situation, and the uncertain results of surgical reconstruction were all taken into account for treatment planning. Following a comprehensive radiological and clinical assessment, prosthetic rehabilitation by a zygomatic implant was suggested due to poor bone quality in the maxillary arch. The intraoral condition is shown in Figure 1, and the radiographic image is shown in Figure 2 with less bone quantity in the maxillary arch. 

**Figure 3:**
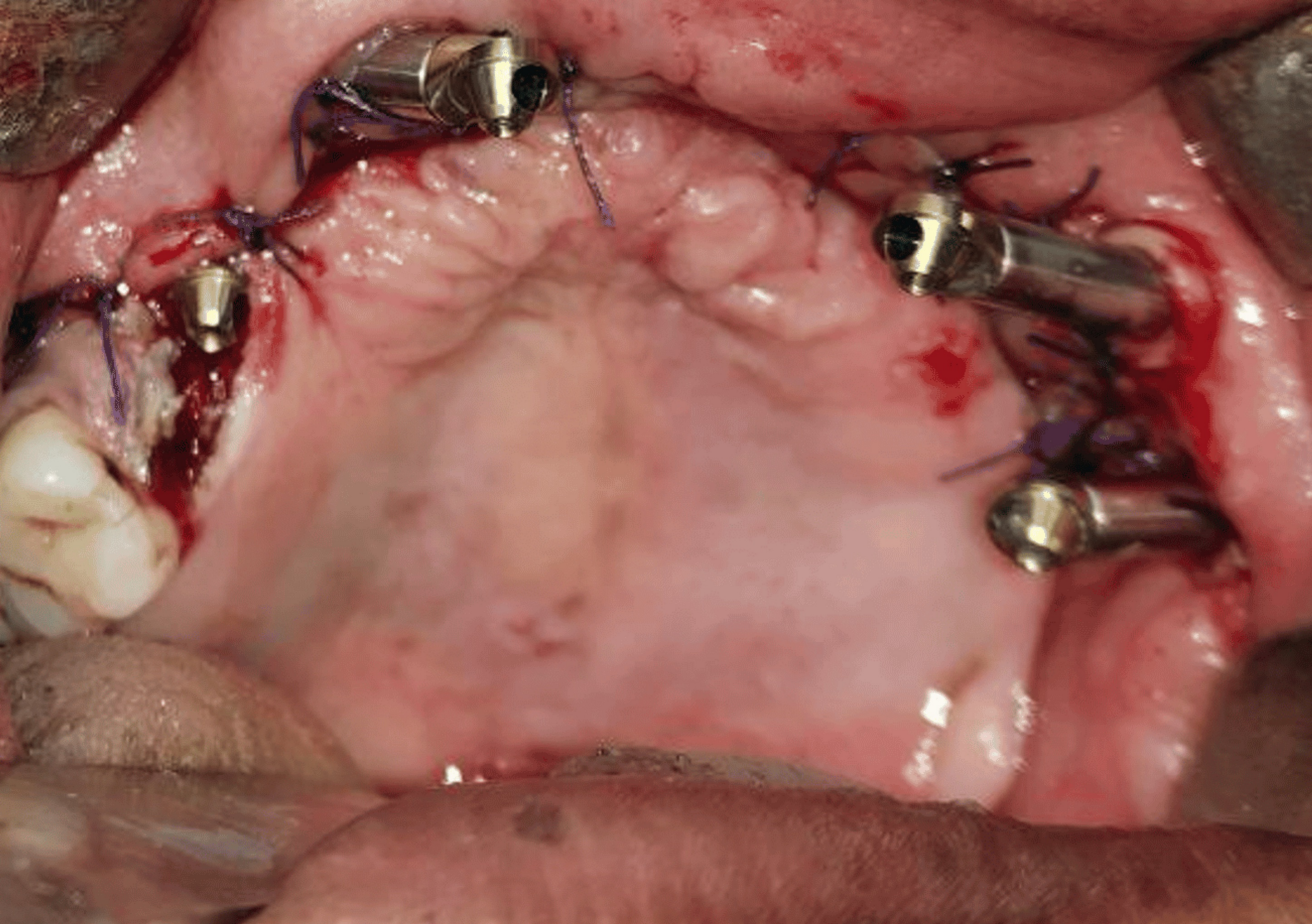
Four zygomatic implants were placed in the maxillary arch

**Figure 4:**
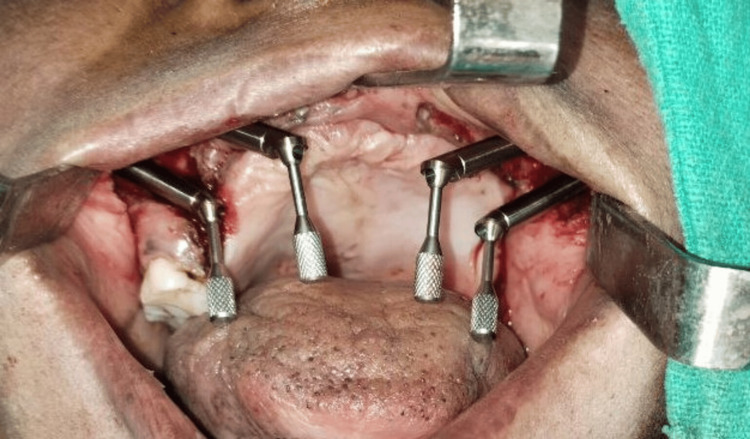
Multiangled abutments attached to quad zygoma implants

Insufficient support framework to stabilize a surgical template necessitated the planning of a freehand surgical operation. The zygoma's body was made visible by a vestibular incision, and both zygomas had osteotomies created. A bilateral quad-zygoma-implant configuration was modified to the placement of zygomatic implants 45 degrees (32.5 mm × 4 mm) (Noris Medical Dental Implant Solutions), two on the right and two on the left side torque achieved. Multiunit abutments (Noris Medical Dental Implant Solutions) were connected to the implants, as shown in Figure 3 and Figure 4.

**Figure 5:**
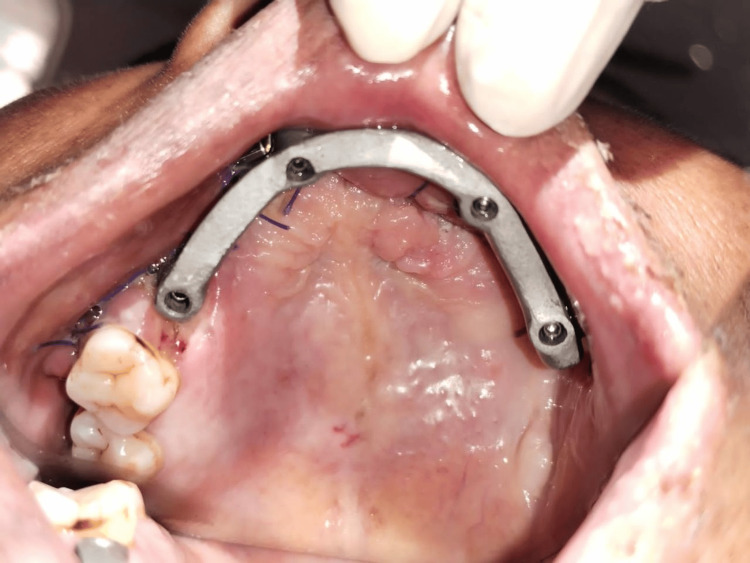
Jig verification done intraorally

After implant placement, intraoral scanning was done to record the positions of the implants. A mock-up framework which was fabricated digitally was attached and was tried intraorally as shown in Figure 5.

**Figure 6:**
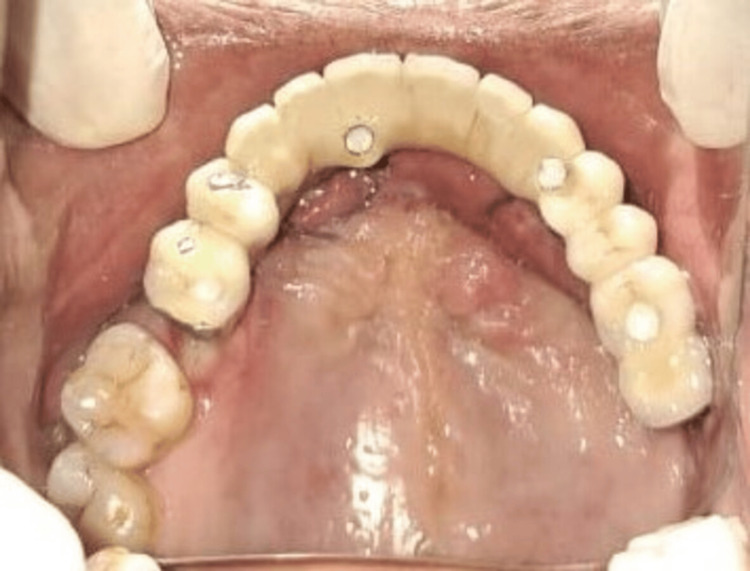
This image shows prosthesis intraorally

**Figure 7:**
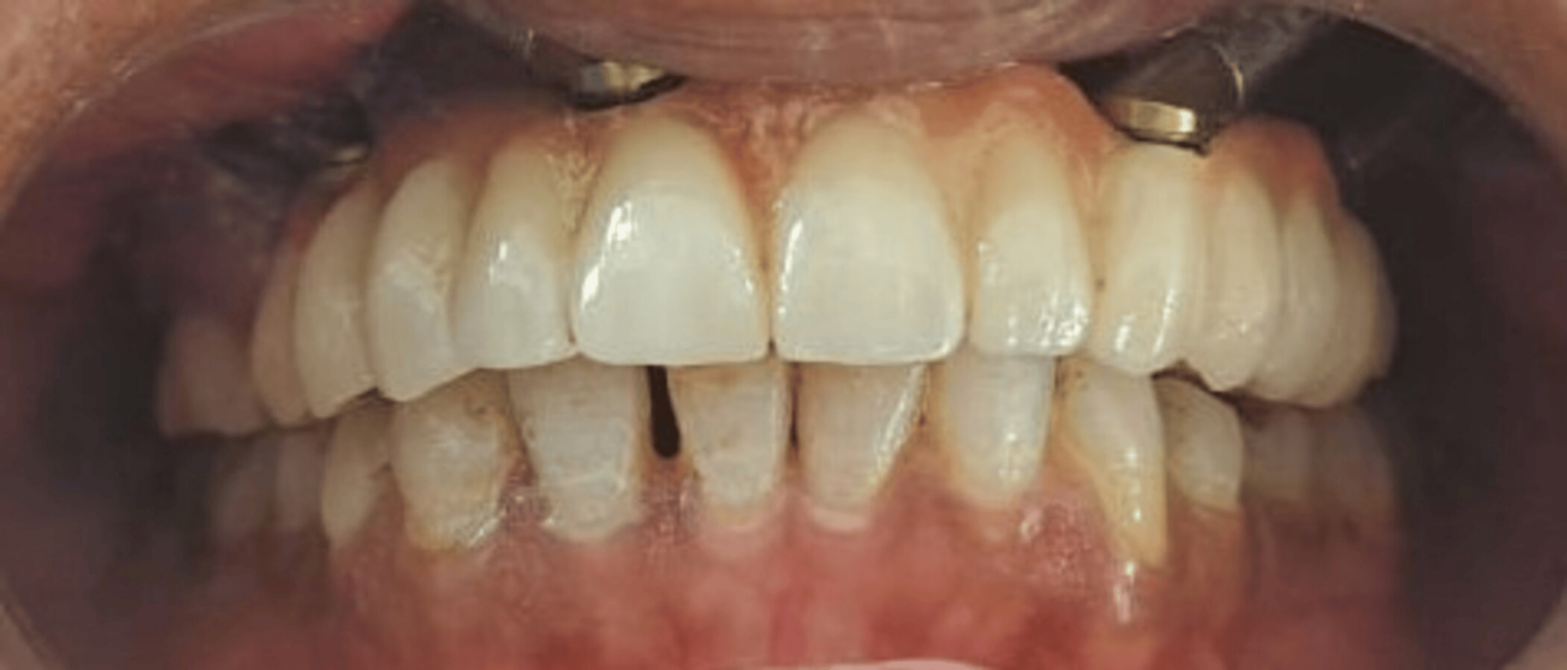
This image shows the maxillary and mandibular arches in occlusion

After measuring the inter-arch distance (11 mm), a fixed prosthesis (FP)-2 type of prosthesis made of porcelain fused to a metal was planned, as shown in Figure 6, and the final photograph of maxillary prosthesis insertion is shown in Figure 7 with proper shade matching.

## Discussion

Long-term success with implant-supported prostheses depends on an understanding of their biomechanics. Implant biomechanics are different from that of native teeth, which have a distinct feedback mechanism for occlusal awareness [9,10]. Following a maxillectomy, zygoma implants are a crucial backup option for secure support when a microsurgically revascularized bone flap restoration is not feasible to achieve quickly. Because of their design, which offers bi-cortical support through the malar bone, zygoma implants can be inserted even in situations where there are complete defects in the maxillary bone. Zygomatic implants have demonstrated better clinical results than bone grafting, which may create a new gold standard procedure for treating compromised maxillary bone [11]. However, Kosaka et al. found no evidence of a substantial drop in salivary cortisol levels for short-term prosthesis wear (subjects who had worn a prosthetic for less than three months) in their research of hemimandibulectomy patients. The variations can be attributed to variations in instances as well as prosthesis types (fixed versus detachable). A detachable prosthesis requires more time to adjust and frequent adjustments throughout the follow-up phase. There have also been reports of a considerable drop in salivary cortisol levels following prosthesis adjustment [12].

According to Rossi et al.'s study, the zygomatic implant stimulated hyperactivity in the muscle fibers during mandibular posture and chewing conditions. This is likely because periodontal receptors, which are important for preparing a bolus for swallowing, are absent from the implant [13]. In 2017, Agliardi et al. reported a prospective six-year follow-up research in which they used both extrasinus and intrasinus procedures, loading the provisional prostheses immediately. No zygomatic implants were lost, yielding a 100.0% success rate [14]. Since the patient in this instance has an intact orbital floor and palate wall and no oroantral connections, additional surgical techniques are not necessary. Zygomatic implants may provide an alternative to bone grafting techniques for maxillary reconstruction as well as obturators that utilize dental implants or not. This can address the issue of inadequate maxillary bony support. The zygoma implant has greatly improved prosthesis retention, which translates into improved stability [15-17]. 

## Conclusions

It is evident that zygomatic fixations have been the subject of numerous investigations conducted in recent years. With advantages like prosthetic rehabilitation of atrophic maxilla and rehabilitation of postresection cases or congenital diseases, graftless protocol with minimal armamentarium and immediate loading can be done, yielding a notable number of clinical cases with prosthetic function. Zygomatic implants are a dependable substitute therapy with a high success rate that is compatible with traditional implants for those who are completely edentulous, especially in patients with post-COVID-19 mucormycosis. So, this treatment option can be considered when bone quantity and quality are not sufficient in the maxillary arch.
